# Normal T and B Cell Responses Against SARS-CoV-2 in a Family With a Non-Functional Vitamin D Receptor: A Case Report

**DOI:** 10.3389/fimmu.2021.758154

**Published:** 2021-09-30

**Authors:** Martin Kongsbak-Wismann, Fatima A. H. Al-Jaberi, Jonas Damgård Schmidt, Mustafa Ghanizada, Cecilie Bo Hansen, Daniel Villalba Lopez, Anders Woetmann, Niels Ødum, Charlotte Menné Bonefeld, Anette Stryhn, Peter Garred, Søren Buus, Carsten Geisler

**Affiliations:** ^1^ The LEO Foundation Skin Immunology Research Center, Department of Immunology and Microbiology, Faculty of Health and Medical Sciences, University of Copenhagen, Copenhagen, Denmark; ^2^ Laboratory of Experimental Immunology, Department of Immunology and Microbiology, Faculty of Health and Medical Sciences, University of Copenhagen, Copenhagen, Denmark; ^3^ Laboratory of Molecular Medicine, Department of Clinical Immunology Section 7631, Rigshospitalet, Copenhagen University Hospital, Copenhagen, Denmark

**Keywords:** COVID-19, vitamin D, HVDRR, adaptive immunity, SARS-CoV-2

## Abstract

The coronavirus disease 2019 (COVID-19) pandemic has severely impacted daily life all over the world. Any measures to slow down the spread of severe acute respiratory syndrome coronavirus 2 (SARS-CoV-2) and to decrease disease severity are highly requested. Recent studies have reported inverse correlations between plasma levels of vitamin D and susceptibility to SARS-CoV-2 infection and COVID-19 severity. Therefore, it has been proposed to supplement the general population with vitamin D to reduce the impact of COVID-19. However, by studying the course of COVID-19 and the immune response against SARS-CoV-2 in a family with a mutated, non-functional vitamin D receptor, we here demonstrate that vitamin D signaling was dispensable for mounting an efficient adaptive immune response against SARS-CoV-2 in this family. Although these observations might not directly be transferred to the general population, they question a central role of vitamin D in the generation of adaptive immunity against SARS-CoV-2.

## Introduction

Recently, it has been reported that vitamin D might affect COVID-19 disease severity. Thus, it has been described that people suffering from vitamin D deficiency have a higher risk of contracting COVID-19 and that vitamin D levels inversely correlate with the disease severity, serum levels of inflammatory markers and fatality rates (1, 2). In line with this, length of hospitalization, intensive care unit admission and mortality have been reported to correlate with vitamin D serum levels (3–10). Based on these and similar studies, it was suggested that vitamin D supplementation of the general population might reduce the viral spread and decrease the disease severity and thereby aid in tackling the SARS-CoV-2 pandemic (1, 3, 5, 6, 10–12). However, recent meta-analyses found that the evidence to support vitamin D supplementation for the prevention or treatment of COVID-19 was inconclusive (13–15). In line with this, a randomized, double-blind, placebo-controlled trial found that vitamin D supplementation did not reduce the overall risk of acute respiratory tract infection in general (16). Thus, whether vitamin D affects the susceptibility to and the course of COVID-19 is still an open question.

Vitamin D is known for its immunomodulatory properties. Vitamin D affects both T cell activation and differentiation (17–19). Vitamin D reduces IFNγ and IL-17 and increases IL-4 and IL-13 secretion (10, 17, 20–22), hence vitamin D is thought to skew immune responses away from T helper (Th) 1/Th17 towards Th2 dominated responses. Importantly, vitamin D exerts its function *via* the vitamin D receptor (VDR) that is expressed by various immune cells (18). The VDR is a member of the nuclear receptor family of transcription factors. Normally, the active form of vitamin D signals by binding to the VDR. Vitamin D/VDR complexes translocate to the nucleus, where they bind DNA and regulate the transcription of vitamin D-responsive genes (23). Interestingly, a recent study found that expression of the VDR was reduced in peripheral blood cells from COVID-19 patients compared to controls especially in male patients (24). We have recently described a patient suffering from hereditary vitamin D resistant rickets (HVDRR) with a novel mutation in the VDR (25). HVDRR is an autosomal recessive disease, which has only been described in ~150 individuals since its first characterization in 1978 (26). In the present HVDRR patient, the VDR mutation locates to the DNA-binding domain of the VDR, completely abolishing the transcriptional activity of the vitamin D/VDR complex. The parents of the HVDRR patient are heterozygous for the mutation, and their T cells exert a 50% reduction in terms of vitamin D responsiveness due to the expression of both the mutated and the wild-type VDR alleles (25). The patient and her parents contracted COVID-19 in the beginning of 2021 and thereby a unique possibility to study the consequences of vitamin D signaling for immune responses against SARS-CoV-2 occurred.

## Case Presentation

A detailed clinical description of the HVDRR patient was recently published (25). In short, the patient was born in 1992 and developed rickets and alopecia within the first year after her birth. The patient was initially treated orally with 1-α-hydroxycholecalciferol (alfacalcidol), calcium and fish oil. The treatment was terminated when she was three years old. In 1999, the patient was referred to hospital due to muscle and bone pains, short stature, and alopecia. At the physical examination, the patient appeared normal except for alopecia and short stature with a height and weight below the 3% percentile. She had normal serum levels of calcium, phosphate, alkaline phosphatase, and parathyroid hormone. However, the level of serum 1,25(OH)_2_D was highly elevated between 320 - 388 pM (normal range 51 – 177 pM), whereas the level of 25(OH)D was normal between 39 – 60 nM (normal range 26 – 150 nM). It was concluded that the patient suffered from HVDRR, and treatment with 600 mg calcium per day and calcitriol (rocaltrol) 0.5 µg twice daily was initiated. Since then, she was regularly seen in the outpatient clinic. She often complained of bone pains in the arms and legs but otherwise she was doing well, and she partially catched up for her low stature, body weight and delayed bone age. The serum levels of 1,25(OH)_2_D were permanently elevated but she kept on having normal serum levels of calcium, phosphate, alkaline phosphatase, and parathyroid hormone. The patient does not have any known COVID-19 comorbidities (27) and has a normal BMI of 24.5 kg/m^2^. In early 2021, the HVDRR patient and her parents contracted SARS-CoV-2 diagnosed by PCR tests of throat swabs. Surprisingly, they all had a mild disease course with fever, nasal congestion, headache, fatigue and anosmia for 3-4 days (**Supplementary Table 1**). As this family represented a unique possibility to study the role of vitamin D signaling in immune responses to SARS-CoV-2, we set out to determine their post-infectious SARS-CoV-2-specific immune response.

## Immune Response

Infection with SARS-CoV-2 normally triggers the immune system to activate and expand SARS-CoV-2 specific CD4^+^ and CD8^+^ T cells and to produce SARS-CoV-2-specific antibodies (28–31). CD4^+^ T cell responses are detected in more than 90% of SARS-CoV-2 infections, whereas CD8^+^ T cell responses are observed in approximately 70% of the cases (32, 33). The best indicator for a mild COVID-19 disease outcome is the existence of SARS-CoV-2 specific CD4^+^ T cells (34). Furthermore, rapid induction of SARS-CoV-2-specific CD4^+^ T cells following infection correlates with a mild disease outcome (35). Conversely, the lack of SARS-CoV-2-specific CD4^+^ T cells is associated with severe or fatal COVID-19 (34, 35). Seroconversion of antibodies against the SARS-CoV-2 spike protein is seen in more than 90% of COVID-19 patients (36, 37). The most immunodominant structure of the spike protein is the host receptor-binding domain (RBD) (38). Neutralizing antibody titers against RBD correlate with COVID-19 disease severity (37, 38); however, so far only limited effects in clinical trials with the injection of SARS-CoV-2 neutralizing monoclonal antibodies have been documented (39). Therefore, it has been proposed that SARS-CoV-2-specific T cells are the main immunological compartment controlling primary SARS-CoV-2 infection (30).

**Figure 1:**
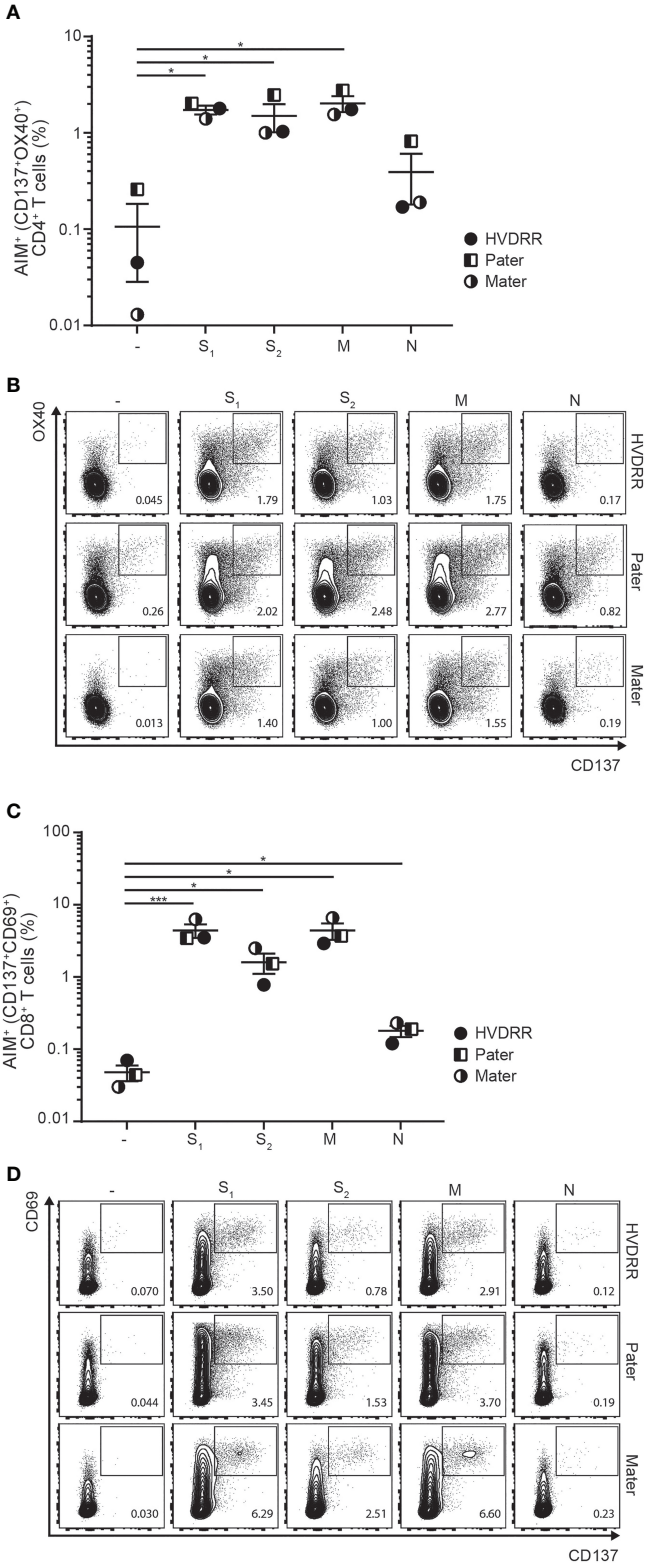
Normal generation of SARS-CoV-2-specific CD4^+^ and CD8^+^ T cells in the HVDRR patient and her parents. **(A)** SARS-CoV-2-specific CD4^+^ T cells measured as percentage of AIM^+^ (CD137^+^OX40^+^) CD4^+^ T cells after stimulation of PBMC with peptide pools encompassing the first half of the spike protein (S_1_), the second half of the spike protein (S_2_), the membrane protein (M) and the nucleoprotein (N). (-) represents control PBMC treated with the vehicle DMSO. Data are shown as mean ± SEM; *p < 0.05. Samples were from the HVDRR patient (filled circles), pater (half-filled squares) and mater (half-filled circles). **(B)** FACS dot plots showing the fraction of AIM^+^ (CD137^+^OX40^+^) CD4^+^ T cells. **(C)** SARS-CoV-2-specific CD8^+^ T cells measured as percentage of AIM^+^ (CD137^+^CD69^+^) CD8^+^ T cells after stimulation of PBMC with peptide pools as described in **(A)**. Data are shown as mean ± SEM. *p < 0.05; ***p < 0.005. Symbols indicate the individuals as described in **(A)**. **(D)** FACS dot plots shoving the fraction AIM^+^ (CD137^+^CD69^+^) CD8^+^ T cells.

First, we determined whether the HVDRR patient and her parents had generated SARS-CoV-2-specific T cells. To identify SARS-CoV-2-specific memory T cells, we stimulated PBMC for 24 hours with overlapping peptides covering the entire SARS-CoV-2 spike (S), membrane (M) and nucleocapsid (N) proteins (for detailed Material and Methods please see **Supplementary Material**). We subsequently measured the number of CD4^+^ and CD8^+^ T cells expressing the activation-induced markers (AIM) CD137/OX40 and CD137/CD69, respectively, as previously defined (28, 32, 34). In all three family members, we found a clear induction of both CD4^+^AIM^+^ (**Figures 1A, B**, for gating strategy please see **Supplementary Figure 1A**) and CD8^+^AIM^+^ cells (**Figures 1C, D**) with frequencies comparable to those previously reported for SARS-CoV-2-specific CD4^+^ and CD8^+^ memory T cells in COVID-19 convalescent individuals (32, 34).

**Figure 2:**
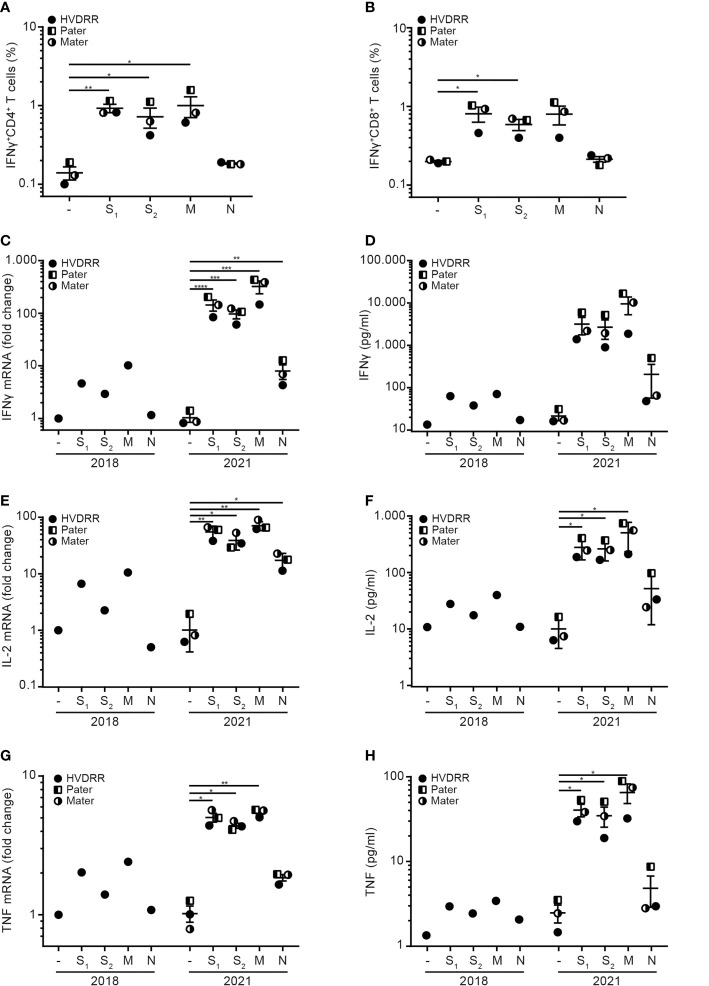
Normal cytokine profile of SARS-CoV-2-specific T cells from the HVDRR patient and her parents. Frequency of IFNγ^+^ **(A)** CD4^+^ and **(B)** CD8^+^ T cells in PBMC treated with DMSO (-) or peptide pools encompassing the first half of the spike protein (S_1_), the second half of the spike protein (S_2_), the membrane protein (M) and the nucleoprotein (N). Samples were from the HVDRR patient (filled circles), pater (half-filled squares) and mater (half-filled circles). Data are shown as mean ± SEM. **(C–H)** Cytokine mRNA and cytokine concentration in the supernatants of PBMC obtained from the HVDRR patient in 2018 and from the HVDRR patient and her parents in 2021 and stimulated as described above. The mRNA expression levels of **(C)** IFNγ, **(E)** IL-2 and **(G)** TNF are shown as geometric mean ± SD. Concentrations of **(D)** IFNγ, **(F)** IL-2 and **(H)** TNF in the supernatants are shown as mean ± SEM; *p < 0.05; **p < 0.01; ***p < 0.005; ****p < 0.001.

T cell responses against SARS-CoV-2 are characterized by secretion of IFNγ, IL-2 and TNF with little or no secretion of IL-17, IL-4 and IL-13 (28, 32, 34). To determine whether vitamin D signaling affected SARS-CoV-2-specific T cell responses, we determined the T cell cytokine profile in PBMC stimulated with SARS-CoV-2 peptides for 24 h. In line with other studies, we found a clear induction of IFNγ^+^CD4^+^ T cells in PBMC stimulated with the spike peptide pools and membrane peptides (**Figure 2A**, for gating strategy please see **Supplementary** **Figure 1B**). Stimulation with peptides from the nucleoprotein did not result in any significant induction of IFNγ^+^CD4^+^ T cells. We observed a similar pattern with generation of IFNγ^+^CD8^+^ T cells in PBMC stimulated with the spike peptide pools and membrane peptides but not with the peptides from the nucleoprotein (**Figure 2B**). Thus, SARS-CoV-2-specific T cells from the HVDRR patient and her parents were clearly able to produce IFNγ.

In 2018, PBMC from the HVDRR patient were isolated and cryo-preserved, which allowed us to compare her T cell responses against the SARS-CoV-2 peptide pools before and after the SARS-CoV-2 infection. In line with the flow cytometry measurements described above, we found that cells isolated post-infection significantly up-regulated gene expression and secretion of IFNγ when stimulated with the S_1_, S_2_ and M SARS-CoV-2 peptide pools, whereas cells isolated in 2018 did not (**Figures 2C, D**). Similar results were seen for IL-2 and TNF (**Figures 2E–H**). Furthermore, we determined the secretion of the Th2 cytokines IL-4 and IL-13 following activation with the SARS-CoV-2 peptides. In contrast to IFNγ, IL-2 and TNF, the SARS-CoV-2 peptides did not induce production of IL-4 and IL-13 (**Supplementary Figures 2A, B**). Thus, the post-infectious SARS-CoV-2-specific T cells isolated from the HVDRR patient, and her parents had a Th1-like cytokine profile as previously described for SARS-CoV-2-specific T cells (28, 32, 34).

**Figure 3:**
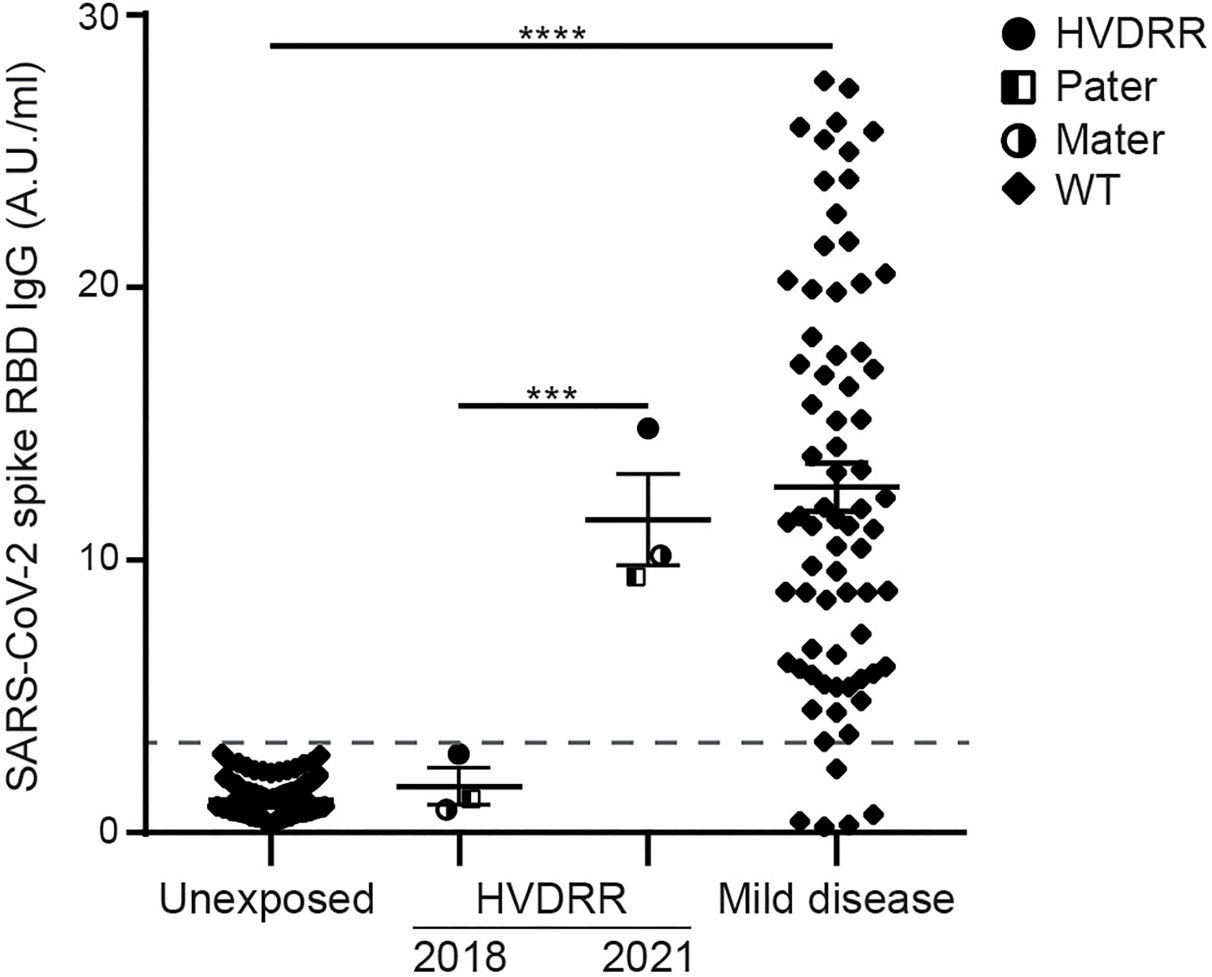
Normal SARS-CoV-2 RBD-specific IgG antibody titers in the plasma of the HVDRR patient and her parents. SARS-CoV-2 RBD-specific IgG antibody titers in the plasma from unexposed subjects (n = 200), subjects with a mild course of COVID-19 (n = 72) and from the HVDRR patient and her parent from 2018 and 21 days after COVID-19 in 2021. The dashed line represents the cut-off for the ELISA (10). Antibody titers are shown for each individual and as mean ± SEM; ***p < 0.005, ****p < 0.001. HVDRR patient (filled circles), pater (half-filled squares) and mater (half-filled circles).

In addition to measuring T cell responses against SARS-CoV-2, we also investigated whether the HVDRR patient and her parents could mount an antibody response against SARS-CoV-2. In order to do this, we analyzed pre-infection plasma samples obtained in 2018 and plasma samples isolated 21 days post symptom onset for IgG antibodies against the RBD of the spike protein. We found that the anti-RBD IgG titers in plasma from 2018 from the HVDRR patient and her parents were similar to the anti-RBD IgG titers in plasma from unexposed healthy individuals (**Figure 3**). Importantly, post-infection plasma from the HVDRR patient and her parents showed similar levels of IgG antibodies against the RBD as plasma from SARS-CoV-2 convalescent in the general population that had had a mild course of COVID-19 (**Figure 3**). These data were compatible with the notion that both the HVDRR patient and her parents mounted a normal humoral immune response against SARS-CoV-2.

## Discussion

In this study, we used a unique human genetic defect to tackle the question of the importance of vitamin D signaling in SARS-CoV-2 infections. The HVDRR patient has a mutation in the DNA-binding domain of the VDR that completely abolishes the transcriptional activity of vitamin D/VDR complexes (25). Thus, cells from the HVDRR patient are unresponsive to vitamin D and she therefore constituted a unique possibility to study the consequences of abolished vitamin D signaling in the immune response against SARS-CoV-2. In addition, studies of her parents, that were heterozygous carriers of the mutated VDR and therefore had a 50% reduction in vitamin D responsiveness (25), could further contribute to the knowledge of the role vitamin D in COVID-19.

The HVDRR patient and her parents contracted COVID-19 in the beginning of 2021, and they all had a mild disease course only lasting 3-4 days. We found that normal numbers of CD4^+^ and CD8^+^ SARS-CoV-2-specific T cells were generated in the HVDRR patient and her parents, and that the cytokine profile of these cells was similar to the cytokine profile of SARS-CoV-2-specific T cells previously described (28, 32, 34). These observations indicated that activation, differentiation and generation of CD4^+^ and CD8^+^ SARS-CoV-2-specific T cells can occur in the absence of vitamin D signaling. Furthermore, we found that the HVDRR patient and her parents generated comparable levels of IgG antibodies against the RBD of the SARS-CoV-2 spike protein as COVID-19 convalescent in the general population that had had a mild course of COVID-19. More studies of the general population are needed to fully understand the role of vitamin D in the immune response against SARS-CoV-2. Thus, vitamin D could play a role in dampening the cytokine storm seen in patients with severe COVID-19 and might also play a role in innate immunity against SARS-CoV-2 for example in generation of cathelicidin (10, 40, 41). We acknowledge, that the current findings in a family with a rare mutation can not necessarily be translated to the general population.

Nonetheless, our findings demonstrate that an efficient adaptive immune response against SARS-CoV-2 can be generated in individuals with reduced (the parents) or no (the patient) vitamin D signaling.

## Data Availability Statement

The raw data supporting the conclusions of this article will be made available by the authors, without undue reservation.

## Ethics Statement

The studies involving human participants were reviewed and approved by The Regional Ethical Committee of the Capital Region of Denmark (H-20028627) and (H-17040922). The patients/participants provided their written informed consent to participate in this study. Written informed consent was obtained from the individual(s) for the publication of any potentially identifiable images or data included in this article.

## Author Contributions

CG, MK-W, and FA-J conceived the study. CG, MK-W, PG, AS, and SB designed the experiments. MK-W, FA-J, JS, MG, CH, and DL performed the laboratory experiments. CB, AW, and NØ assisted with the experimental design and data interpretation. CG, MK-W, and FA-J analyzed the data and wrote the manuscript with input from all authors. All authors contributed to the article and approved the submitted version.

## Funding

This work was financially supported by grants from the Danish Council for Independent Research (8020-00066B) to CG and the Carlsberg Foundation (CF20-0045) and the Novo Nordisk Foundation (NFF205A0063505 and NNF20SA0064201) to CH and PG.

## Conflict of Interest

The authors declare that the research was conducted in the absence of any commercial or financial relationships that could be construed as a potential conflict of interest.

## Publisher’s Note

All claims expressed in this article are solely those of the authors and do not necessarily represent those of their affiliated organizations, or those of the publisher, the editors and the reviewers. Any product that may be evaluated in this article, or claim that may be made by its manufacturer, is not guaranteed or endorsed by the publisher.
