# Supplementary material for: Normal T and B Cell Responses Against SARS-CoV-2 in a Family With a Non-Functional Vitamin D Receptor: A Case Report
Source: Front Immunol. 2021 Sep 30;12:758154. doi: 10.3389/fimmu.2021.758154 (PMC8515133; doi:10.3389/fimmu.2021.758154)
Supplement: Supplementary file 1 [file DdataSheet_1.pdf]

Supplementary Material

Normal T and B cell responses against SARS-CoV-2 in a family with a non-functional vitamin D receptor: A case report

Materials and Methods

PBMC purification and peptide stimulation

All procedures involving the handling of human samples are in accordance with the principles described in the Declaration of Helsinki and control samples were collected and analyzed according to ethically approval by the Regional Ethical Committee of the Capital Region of Denmark (H-20028627). The samples from the index case and her parents were obtained with written informed consent and the use is approved by Regional Ethical Committee of the Capital Region of Denmark (H-17040922). Mononuclear cells from donor blood were isolated by Lymphoprep (Axis Shield from Oslo, Norway) density gradient centrifugation by using SepMate^{TM} tubes (85450, Stemcell Technologies, Canada). Purified peripheral blood mononuclear cells (PBMC) were cultured at a concentration of 5 x 10^6 cells/ml in serum-free X-VIVO 15 medium (BE02-060F, Lonza, Verviers, Belgium) for 24 h in round-bottomed 96-well tissue culture plates (163320, Nunc, ThermoFisher Scientific, MA, USA). For stimulation, PBMC were pre-treated with a co-stimulatory mix of anti-human CD28/CD49d antibodies (347690, BD Biosciences, USA) for 2 h before the addition of peptide pools. Peptide pools were 15-mers with an 11 amino acid overlap (Genscript, NJ, USA) spanning the SARS-CoV-2 spike glycoprotein subpool 1 (S_1) and subpool 2 (S_2) (RP30020), membrane protein (RP30022) and nucleoprotein (RP30013). All peptide pools were dissolved in DMSO and used in a final concentration of 0.5 μg/ml. In experiments aimed at detecting of IFNγ^+ T

cells, cell cultures were supplemented with Brefeldin A (B7651, Sigma-Aldrich, MO, USA) in a final concentration of 0.5 µg/ml.

Flow cytometry

For surface staining, a mix of antibodies (Antibody panel 1), was prepared in brilliant stain buffer (566349, BD Biosciences, CA, USA), and 1 x 10^6 wells were stained in a volume of 50 µl for 30 min at 4 ºC. Subsequently, cells were washed twice in 200 µl PBS and analyzed on a Fortessa 5 laser flow cytometer (BD Biosciences). For intracellular staining, cells were initially surface stained as described above with a mix of antibodies (Antibody panel 2). Subsequently, cells were stained with anti-IFNγ using the FoxP3 staining kit (00-5523-00, ThermoFisher Scientific) according to the manufacturer's instructions.

Antibody panel 1<br>Antigen	Clone	Fluorochrome	Catalog#	Vendor
CD3	UCHT1	BV421	562426	BD Biosciences
CD4	RPA-T4	PerCP-Cy5.5	560650	BD Biosciences
CD8	RPA-T8	BV786	563823	BD Biosciences
CD69	FN50	PE	310906	BioLegend
CD137	4B4-1	APC	561702	BD Biosciences
CD134/OX40	L106	BV650	745372	BD Biosciences

Antibody panel 2<br>Antigen	Clone	Fluorochrome	Catalog#	Vendor
CD3	UCHT1	BV421	562426	BD Biosciences
CD4	RPA-T4	PerCP-Cy5.5	560650	BD Biosciences
CD8	RPA-T8	BV786	563823	BD Biosciences
IFNγ	4S.B3	FITC	552887	BD Pharmingen

RT-qPCR

mRNA levels for various targets were measured by RT-qPCR. Following cell isolation, cells were lysed in TRI Reagent (T9424, Sigma-Aldrich) and mixed with the phase separation reagent 1-bromo-3-chloropropane (B9673, Sigma-Aldrich). The RNA phase was isolated and mixed with

isopropanol supplemented with glycogen for RNA precipitation (10814-010, Invitrogen, ThermoFischer Scientific). The RNA pellet was then washed in RNase free 75% ethanol 3 times. cDNA was synthesized from quantified RNA using High-Capacity RNA-to-cDNA™ Kit (4387406, Applied Biosystems ThermoFischer Scientific) according to the manufacturer's instructions. For RT-qPCR, 12.5 ng cDNA was mixed with TaqMan® Universal Master Mix II with Uracil-N glycosylase and the target primers. We used the following primers from Applied Biosystems: β-actin (Hs_ 01060665_g1), IFNγ (Hs00989291_m1), IL-2 (Hs00174114_m1), TNF (Hs01113624_g1). The plate-based detection instrument LightCycler ® 480 II from Roche was used for RT-qPCR. Cytokine expression levels were determined relative to the expression levels of β-actin.

MSD ELISA

Following stimulation with peptide pools for 24 h, supernatants were isolated and stored at -80 °C. Subsequently the supernatants were analyzed for cytokines using the V-PLEX Proinflammatory Panel 1 Human Kit (K15049D, Meso Scale Diagnostics, Maryland, USA). Supernatants were incubated in plates with pre-coated wells with capture-antibodies. The plates were washed and incubated with capture antibodies wee against IFNγ, IL-2, IL-10, IL-4, IL-12p70, IL-6, IL-13, IL-8, IL-1β and TNFα for 2 hours. The plates were washed again and incubated with secondary SULFO-TAG detection antibodies for 1 h. Finally, the plates were analyzed using the MESO QuickPlex SQ 120 and Discovery Workbench® v4.0.

Anti-SARS-CoV-2 receptor binding domain (RBD) ELISA measurements

Antibodies specific for the RBD of the SARS-CoV-2 spike protein was detected as previously described (10). In short, 96-well Nunc MaxiSorp plates were coated with purified RBD in PBS over

night at 4 ºC. Wells were blocked for 1 h with PBS-T before addition of plasma samples, which allowed potential anti-RBD antibodies to bind. Next, HRP-conjugated polyclonal antibodies against human IgG were added. Subsequently, TMB One substrate was applied and allowed to react for 7 min before the reaction was stopped using H_2SO_4. OD was measured at 450-630 nm using a Synergy HT absorbance reader.

Quantification and statistical analysis

Student's t-tests (paired, one-tailed) were used to determine statistical significance in all experiments including the HVDRR family members. Student's t-test (unpaired, one tailed, equal variance) was used to determine significance in the IgG antibody response measurements. Significance levels are as follows: * $p < 0.05$; ** $p < 0.01$; *** $p < 0.005$; **** $p < 0.001$. Data are presented as mean values with SEM (FACS and MSD ELISA analysis) or geometric mean with SD (RT-qPCR analysis) as indicated.
