# Supplementary material for: Normal T and B Cell Responses Against SARS-CoV-2 in a Family With a Non-Functional Vitamin D Receptor: A Case Report
Source: Front Immunol. 2021 Sep 30;12:758154. doi: 10.3389/fimmu.2021.758154 (PMC8515133; doi:10.3389/fimmu.2021.758154)
Supplement: Supplementary file 2 [file Image_1.pdf]

Supplementary Figure 1
Kongsbak-Wismann et al.

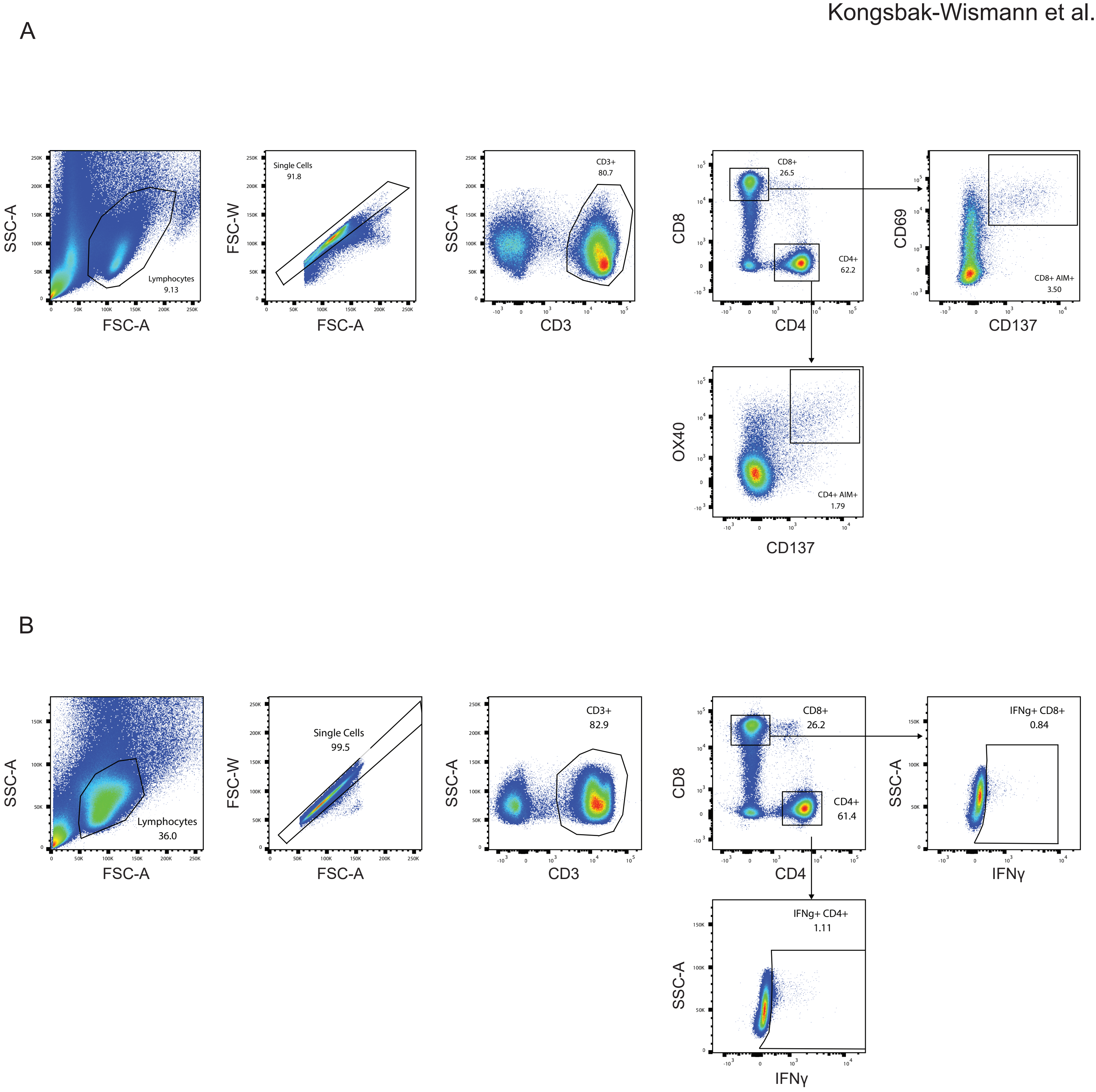

(A) Gating strategy for detection of the activation-induced markers (AIM) CD137/OX4 on CD4^{+} T cells and CD137/CD69 on CD8^{+} T cells. **(B)** Gating strategy for detection of IFNγ^{+}CD4^{+} and IFNγ^{+}CD8^{+} T cells.
