# Supplementary material for: Normal T and B Cell Responses Against SARS-CoV-2 in a Family With a Non-Functional Vitamin D Receptor: A Case Report
Source: Front Immunol. 2021 Sep 30;12:758154. doi: 10.3389/fimmu.2021.758154 (PMC8515133; doi:10.3389/fimmu.2021.758154)
Supplement: Supplementary file 3 [file Image_2.pdf]

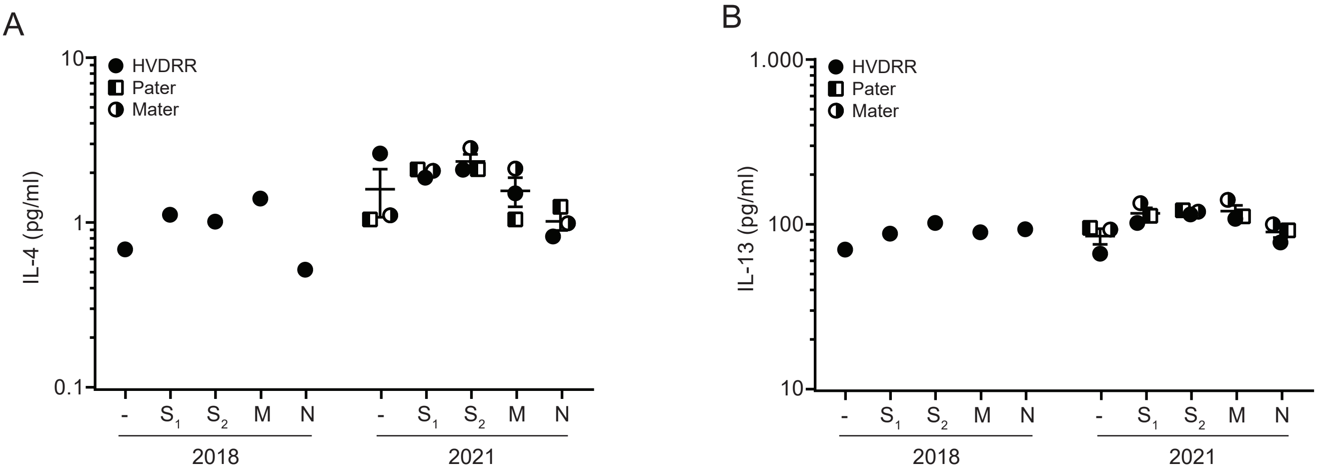

(A) IL-4 and **(B)** IL-13 concentrations in the supernatants of PBMC treated with DMSO (-) or peptide pools encompassing the first half of the spike protein (S1), the second half of the spike protein (S2), the membrane protein (M) and the nucleoprotein (N) obtained from the HVDRR patient in 2018 and from the HVDRR patient and her parents in 2021. Data are shown as mean ± SEM; HVDRR patient (filled circles), pater (half-filled squares) and mater (half-filled circles).
