# Supplementary material for: Normal T and B Cell Responses Against SARS-CoV-2 in a Family With a Non-Functional Vitamin D Receptor: A Case Report
Source: Front Immunol. 2021 Sep 30;12:758154. doi: 10.3389/fimmu.2021.758154 (PMC8515133; doi:10.3389/fimmu.2021.758154)
Supplement: Supplementary file 4 [file Table_1.pdf]

Short characterization of the patients and their COVID-19 related symptoms			
	**HVDRR patient**	**Pater**	**Mater**
Age (years)	29	64	66
Gender	Female	Male	Female
Type of SARS-CoV-2 diagnosis	PCR	PCR	PCR
Sample collection	March 2018<br>February 2021	April 2018<br>February 2021	April 2018<br>February 2021
Symptoms			
**Fever**	x	x	x
**Nasal congestion**	x	x	x
**Pharyngalgia**	-	-	-
**Coughing**	-	x	-
**Shortness of breath**	-	-	-
**Headache**	x	x	x
**Chest pain**	-	-	-
**Muscle and joint pain**	x	-	x
**Fatigue**	x	x	x
**Loss of appetite**	-	-	-
**Nausea**	-	-	-
**Vomiting**	-	-	-
**Diarrhea**	-	-	-
**Abdomial pain**	-	-	-
**Loss of sense of taste/smell**	x	x	x
Duration of illness* (days)	3.5	3	3

*Excluding anosmia
